# To Farm or Not to Farm? Pilot Testing a Sentiocentric Ethical Framework for Farming Non-Typical Species

**DOI:** 10.3390/ani16101519

**Published:** 2026-05-15

**Authors:** Helena Hale, Selene S. C. Nogueira, Sérgio Nogueira-Filho, Adroaldo Zanella, Nicola Rooney, Jessica Bell Rizzolo, Suzanne D. E. Held, Michael Mendl, Siobhan Mullan

**Affiliations:** 1 Animal Welfare and Behaviour Research Group, Bristol Veterinary School, University of Bristol, Bristol BS40 5DU, UK; helena.hale@bristol.ac.uk (H.H.); nicola.rooney@bristol.ac.uk (N.R.); suzanne.held@bristol.ac.uk (S.D.E.H.); mike.mendl@bristol.ac.uk (M.M.); 2 Departamento de Ciências Biológicas, Universidade Estadual de Santa Cruz, Ilhéus 45662-900, BA, Brazil; selene@uesc.br; 3 Departamento de Ciências Agrárias E Ambientais, Universidade Estadual de Santa Cruz, Ilhéus 45662-900, BA, Brazil; 4 Department of Preventive Veterinary Medicine and Animal Health, University of São Paulo, Pirassununga 13635-900, SP, Brazil; adroaldo.zanella@usp.br; 5 Department of Fisheries, Wildlife and Conservation Sciences, Oregon State University, Oregon, OR 97331, USA; jessica.b.rizzolo@gmail.com; 6 School of Veterinary Medicine, University College Dublin, D04 V1W8 Dublin, Ireland

**Keywords:** wildlife, farming, ethics, ethical framework, welfare

## Abstract

Systems that farm non-typical (wild) species for human consumption are on the rise globally, in contrast to more typical livestock production. In some instances, wildlife farming may arguably help alleviate poverty, provide sustainable animal protein, and be a useful strategy for conservation through reducing wildlife poaching or breeding some animals on farms for reintroduction. However, it is unclear whether farming non-typical species within variable and often unregulated systems truly offers these benefits or outweighs the costs including animal welfare implications, public health concerns, and normalising or intensifying the consumption of wild animals. A previous study proposed a sentiocentric ethical decision-making framework for the farming of wild species. In the present study we invited academic ‘key informants’ with specialised knowledge about farming non-typical species to pilot the framework via an online survey using a species of their choice and requested their feedback on its strengths and weaknesses. Thirteen respondents applied ten different mammalian, reptilian, insect, and avian species to the framework, spanning all continents. Ultimately, the framework outcome for 11 appraisals was that the chosen species may be suitable for farming. However, erroneous responses were likely in places, and there was some uncertainty over definitions of framework terminology. We publish resultant amendments to the ethical framework to clarify meaning and suggest that it can be applied proactively or reactively by different stakeholders (e.g., governments, businesses, and NGOs). We reflect our informants’ views, acknowledging the need to solicit expertise from additional stakeholders (e.g., farmers) and the role of cultural significance and rural communities when considering farming non-typical species.

## Simple Summary

Farming non-typical (wild) species for human consumption is on the rise globally as an alternative to typical livestock production. It is argued that this practice may help alleviate poverty, provide sustainable animal protein, and be a useful strategy for the conservation of some species through reducing wildlife poaching or breeding for reintroduction. However, it is unclear whether farming non-typical species truly offers such benefits. An ethical decision-making framework to assess the acceptability of farming wild species was previously constructed, and this study asked for the views of academics in applying the framework to their species of expertise through an online survey. Thirteen responses were received about ten different mammalian, reptilian, insect, and avian species, spanning all continents. Ultimately, the framework outcome for 11 appraisals was that the chosen species may be suitable for farming. However, some responses appeared to arise from misunderstandings of the framework, and there was uncertainty over the definition of the term ‘sentience’. We propose amendments to the ethical framework to clarify meaning and suggest that the framework can be applied by different stakeholders (e.g., governments, businesses, and NGOs). We acknowledge the value of expertise from practical experience as well as scientific knowledge, and the role of cultural significance and rural communities when considering farming non-typical species.

## 1. Introduction

Localised farming of non-typical domestic, semi-domestic, and/or wild species is a long-standing traditional practice for meeting people’s daily needs, especially in agriculturally underdeveloped countries. However, the number of wild animals being farmed globally is reportedly rising [1], with the true numbers and implications of increasingly commercial farming practices remaining non-transparent. Using publicly available information via the published literature and Freedom of Information (FOI) requests, an estimate of the number of wild animals being farmed globally has recently been made to reveal the extent and possible impact of this industry over the past 20 years [2]. At least 487 wildlife species and nearly 1 billion individual wild animals were found to be farmed globally in this period, comprising 27 amphibian, 133 reptilian, 249 avian, and 79 mammalian species, with at least 90 countries having wild vertebrate animal commercial breeding operations [2]. The authors suggest that the accurate number of farmed wildlife is likely to be far higher, due to the paucity of publicly available information and the difficulties they faced obtaining data from the relevant authorities worldwide [2]. When considering the impact of these farms, it is important to note that of the documented species, 34% are listed as either Near Threatened, Vulnerable, Endangered, or Critically Endangered by the IUCN Red List of Threatened Species, and 62% are listed in the appendices of the Convention on International Trade in Endangered Species of Wild Fauna and Flora (CITES) [2].

Commercial farming of non-typical species is encouraged by laws and legislation in many countries (e.g., China, Vietnam, Nepal, and South Africa [2,3]), with numerous terrestrial and aquatic species having been proposed for farming expansion [4]. The interest and support from governmental and non-governmental agencies is also made clear by funded programmes into suitability for farming for sustainable food security (e.g., the EU DIVERSIFY fisheries project [5]). Whilst some species are farmed primarily for human sustenance, for others, this is secondary to the production of leather or feathers for luxury fashion (e.g., crocodilians and ratites), programmes for captive breeding for wildlife conservation efforts alongside meat production (e.g., green sea turtles (*Chelonia mydas*), yacare caiman (*Caiman crocodilus yacare*), Amazonian turtle (*Podocnemis expansa*)) or preventing hunting in the wild [6]. Other species are not farmed for meat, but their parts are used in traditional Chinese medicine (e.g., bears and tigers, [2]). Perceived potential benefits of commercially farmed non-typical species as an alternative to traditional farming systems include aiding poverty alleviation [7], meeting consumer demand while reducing pressure on wild populations [6,8], optimising economic profits [2] and increasing sustainability by being better suited to the local environment, climate, and food [9,10].

However, some potential problems for farming wild species include animal welfare and public health implications, and that farming may not necessarily relieve pressure on wild populations for various reasons [2]. For example, poachers may be further incentivised by increasing the economic value and market preference for wild-caught meat, farmed populations may be restocked with wild animals, and farmed animals may be laundered for the illegal wildlife trade [1,11]. There is only a small body of research into animal welfare in wild farmed species [1,12,13,14,15], with this remaining extremely limited on a global scale and lacking for most species. Furthermore, there is little in the literature regarding the impact of farming non-typical species in relation to public health concerns (e.g., the potential for transmission of novel zoonotic diseases [16]), and the cost–benefit implications for conservation and biodiversity. Vietnam is one of the global hotspots for the farming of hundreds of wild species, including those categorised at the highest level of protection by the Convention on International Trade in Endangered Species of Wild Fauna and Flora (CITES) [17]. However, there is a recognised lack of understanding about the economic incentives of local producers and rural communities, where plugging this gap may aid policy development for addressing a range of priorities including conservation goals, zoonotic disease risk, rural development, and human welfare [18].

Given the paucity of research relating to the appropriateness of farming non-typical species, a preliminary decision-tree framework was developed by Mullan et al. [4], designed to be applied by different stakeholders, to examine the legitimacy of arguments for, and ethical acceptability of, farming non-typical, wild species for human sustenance. The framework uses a utilitarian, sentiocentric approach and is therefore overarchingly concerned with the likelihood of sentience, “the capacity, and level of awareness and cognitive ability, necessary to have feelings” [19], and the impact of being farmed on wild sentient animals as well as wider environmental and human effects. It follows five key steps, and the user is guided through these with suggested additional considerations, as outlined in detail in the original publication [4]. It is important to note that the sentience and welfare needs of typically-farmed animals such as domesticated galliformes, bovine, ovine, and porcine species are widely recognised yet do not preclude current industrialised farming practice or its acceptability. The same can be said for environmental issues and the impact of farming these species on human health. However, applied research into improving typical farming systems can inform positive change for sustainability and welfare. Gathering knowledge of such issues surrounding the farming of non-typically farmed species is promoted by the proposed framework, with the authors conceding that “that there are likely to be few species with no negative consequences so an evaluation of the degree of likely impact is required in order to decide whether it could be acceptable” [4]. The framework also questions whether instances of farming sentient wild species on a small-scale could alleviate an exceptional environmental or human ethical concern (e.g., species conservation, poaching, malnourishment or severe poverty) even when welfare is questionable. Ultimately, the framework aims to guide the user to an outcome about whether the species in question *should* currently be deemed suitable or unsuitable for farming on ethical grounds, and to identify existing gaps in knowledge and practice. We see the framework as a useful tool in contributing towards ongoing discussions and outcomes of farming non-typical species, complementing research into species-specific farming systems and welfare needs.

We recognise that a framework such as this should evolve in light of the feedback around its use and validity with different stakeholders. The present exploratory study aimed, for the first time, to pilot the proposed framework [4] to investigate its construct validity, strengths, and weaknesses for addressing ethical challenges that may aid decision-making on whether a species is suitable for farming. We aimed to solicit the views of academic ‘key informants’ [20], with knowledge in the field of farming non-typical species for human consumption. As summarised by Rizzolo et al. [1], key informants have access to specialised knowledge about a community and/or topic [20]. Collectively, specialised key informant knowledge is useful to gather rich information and deepen understanding. We then aimed to understand the experience of the experts in using the framework in order to enable refinement where necessary prior to future revisions and/or applications with other stakeholders and species.

## 2. Materials and Methods

An online survey of academic key informants’ views of farming non-typical species for food was conducted between June and August 2024. Ethical approval was granted by the UCD Human Research Ethics Committee, approval number: LS-LR-24-207-Mullan. Informed consent was obtained from all subjects involved in the study.

### 2.1. Survey Participants

Target respondents were people with academic knowledge of farming non-typical wild species for food, and they were mostly identified through being an author of a relevant peer-reviewed scientific publication. Google Scholar was used to search for peer-reviewed publications about species-specific wildlife farming, with inclusion criteria being that the paper had been published within the past 10 years, the species was being farmed for food, either directly, or indirectly (e.g., through animal feed), and was considered non-typical (i.e., not currently widely farmed). Alternative purposes for farming (e.g., wildlife conservation, luxury cosmetics or fashion, traditional medicine or the pet trade) could co-occur but were not the sole or main farming output. Target research species and search terms included the farming of various non-typical mammals, fish and other aquaculture, insects, reptiles, and birds as well as general searches for ‘wildlife farming’. Forty-three corresponding authors of peer-reviewed research publications were invited to participate via their publicly available email addresses and provided with a participation information document. A single follow-up email was sent. Furthermore, if the authors of the current study had additional relevant research contacts who met the inclusion criteria but had not been found via the online search, these academics were also invited to participate (n = 10). In total, fifty-three academics were directly contacted regarding survey participation. Contacted academics were also invited to share the survey email with any colleagues involved in researching wildlife farming to enable snowball sampling.

For those who chose to participate, answers were anonymous, and it was possible for respondents to complete the survey more than once for different focal species. At the end of the survey, respondents were given the option to share their contact details for future collaboration. This personal information was not linked to the survey answers to retain anonymity.

### 2.2. Survey Design

The survey was designed and distributed using Jisc Online Surveys v3 (Bristol, UK) and can be found in the Supplementary Materials. The initial items of the survey related to participation consent, which was required before the respondents could progress. Thereafter, the respondents were asked to provide information about their research discipline, length of time researching the farming of non-typical species, geographical region of research, and a farmed wild species of their choice (and research expertise) for the survey questions. They were then asked for background information about their chosen species, including the continent(s) and country(ies) where currently farmed, the scale of farming (increasing, decreasing, static), and the number of farms and total number of animals typically being kept and slaughtered globally each year. Respondents were asked to describe the type of farming system (e.g., enclosure design, husbandry information, slaughter method) and to select the prevalent drivers for the farming of their chosen species (economic, food security/human sustenance, fashion industry, conservation, cosmetic or medicinal products, cultural factors, organised wildlife crime, other), and to describe their answer if they selected ‘other’.

The next set of questions related to the ethical framework for farming non-typical species [4], with an image of the framework provided at the top of each page for reference (the framework, complete with amendments resulting from this study, is shown later in the Results section). Respondents mostly experienced Steps 1 to 5 as they appear in the framework, with the survey designed to skip to the next step depending on their answer. However, for Step 2, if the respondents reported that they did not think there was sufficient biological knowledge to estimate the impact of farming, they were allowed to continue to answer subsequent questions as though there was. This was to maximise the feedback on the subsequent questions, and also in recognition that the species was already being farmed and to allow the respondents to provide their best evaluation of the situation. Relevant considerations that might be taken into account were included with each framework question, as outlined in the original research paper [4] (see the Supplementary Materials). After providing ‘Yes/No’ answers at each step of the framework, respondents were given the option to explain their choice with an open text response. They were also asked to provide any additional considerations or missing components that they felt would be important to guide people through each step of the framework that they experienced.

Once the respondents had completed items relating to the framework, they were advised that they had reached the end of these questions either because the species should not currently be considered suitable for farming, or that the species could be acceptable for farming. However, it was not possible for the respondents to be given their framework outcome, due to constraints of the survey design platform. They were then asked to provide additional feedback via further free text questions, which included how applicable they considered the framework to be across cultures, how helpful they considered the farming of their chosen non-typical species to ensuring human food security, whether there was anything additional that they wished to see in the framework, and if there was anything that they considered redundant in the framework, with the option to explain each of their answers. Respondents were asked if they could identify any specific examples relating to Step 3a in the framework, where in certain limited circumstances, animal welfare could be significantly compromised for a greater benefit, as the research group considered this as an area of particular interest and challenge. Furthermore, respondents were asked if they were aware of any other non-typical species being farmed that may not yet be described in the peer-reviewed literature and to provide details if so, including whether they thought this species would be suitable or unsuitable for farming according to the ethical framework. Finally, free text space was provided for the respondents to feedback on anything additional in relation to their own experience of wildlife farming that had not been covered in the survey. The survey was piloted by members of the research team, resulting in minor changes in question wording and order.

### 2.3. Data Analysis

The data were curated and reported descriptively. Free text responses were summarised, but due to the small number of respondents and relatively short responses to specific questions, thematic analysis was not conducted.

## 3. Results

### 3.1. Respondent Information

Thirteen responses were received, with details of the respondents summarised in Table 1. To the authors’ knowledge, only one respondent completed the survey twice about two snake species, as they referred to the other species in each response. A range of self-described disciplines were provided, most responses were from people with 10+ years’ experience (10/13), and almost all continents were represented. Due to the possibility for snowball sampling, we were unable to obtain more information about the respondents from the information we had about those who we invited directly.

### 3.2. Background Information About Focal Species

The respondents’ chosen focal species for the survey were ten different mammalian, reptilian, insect, and avian species: capybara (*Hydrochoerus hydrochaeris*), collared peccary (*Dicotyles tajacu*), agouti (genus *Dasyprocta*), American bison (*Bison bison*), Burmese python (*Python bivittatus*), Oriental rat snake (*Ptyas mucosa*), scorpion mud turtle (*Kinosternon scorpioides*), crocodilian (species not specified—there are 28 Crocodilia species), black soldier fly (*Hermetia illucens*), and ostrich (*Struthio camelus*). Despite invitations to researchers in the field, aquaculture species were not represented. Three species—capybara, collared peccary and black soldier fly—were discussed by two respondents each. Rather than considering these as duplicate responses, data were retained and are referred to as Respondent 1 and Respondent 2 for each species, since different perspectives were offered about the farming of these species. Furthermore, crocodilian species were discussed collectively, despite respondents having been asked to select one species. This response was also retained as it provided good insight into the farming of crocodilians with some specific examples. Background information provided by each respondent about their chosen species is summarised in Table 2.

**Figure 1:**
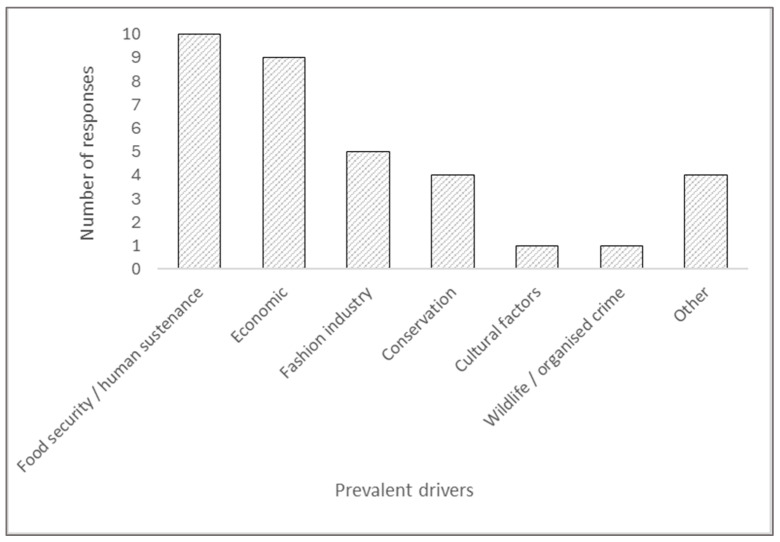
A summary of the prevalent drivers identified by the respondents for the farming of their chosen non-typical species.

Most respondents perceived ‘Food security/human sustenance’ as a prevalent driver for farming their chosen species, with the frequency of all drivers shown in Figure 1. Those who suggested ‘other’ drivers for farming were both black soldier fly (BSF) respondents, who stated that the primary reason for farming BSF was animal feed. The ostrich respondent who suggested a driver for farming was to support the rural economy, and capybara Respondent 1 who said farming this species helped manage human–animal conflicts with increasing numbers in the wild.

The fewest farms were estimated for the scorpion mud turtle, with an estimated 1–10 farms (500–1000 animals kept in total and 60–100 slaughtered each year). In contrast, the highest number of farms were for American bison and Oriental rat snakes, each with 500+ farms (500,000–1,000,000 bison were estimated to live across all farms, with 50,000–100,000 slaughtered annually). For black soldier flies, 100–500 farms were approximated by both respondents, and the number of flies across farms was reported as 1 million+ by Respondent 2 (maximum survey option), whilst Respondent 1 considered a minimum of 8–16 billion were in farms at any given time, and that 190 billion were slaughtered annually in this rapidly growing industry.

Of the 10 species, only capybara farming was reported as in decline (by both respondents), and the farming of agouti, American bison, scorpion mud turtles, crocodilian species and black soldier fly was said to be increasing (both respondents agreed about the latter). For the non-typical species discussed in this survey, farming was reported to occur mostly in their country or continent of origin. Respondents provided free-text responses about the farming systems used for their chosen species, often providing long and detailed responses. Collared peccaries were reportedly frequently farmed in semi-captive environments, managing them in areas of their natural habitat, and this sometimes occurred for capybaras. Alternatively, capybaras are also farmed in paddocks and collared peccaries in intensive settings. Ostrich and American bison were described as typically being farmed in free range systems, albeit with some phases involving more intensive management. Conversely, agoutis were reported to be farmed intensively in cages/pens. Both snake species were described in intensive conditions, and similarly, scorpion mud turtles were reportedly farmed in little space. Given the range of crocodilian species, a variety of farming styles were described, including group housing and individual pens. The black soldier fly larvae were reported as farmed in trays, fed a variety of foods, and larvae killed in a range of ways, including boiling and grinding, depending on the final use.

### 3.3. Application of the Framework

**Figure 2:**
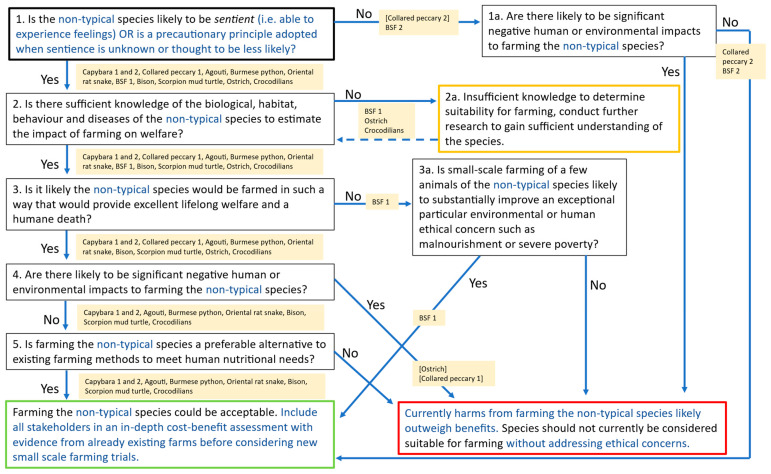
The ethical framework with revisions (in blue text) made in response to survey feedback and the step-by-step answers given per respondent for their chosen species. [Brackets] indicate where the answer selected appeared erroneous based on the respondent’s written justification. BSF = black soldier fly.

The outcome of putting each species through the framework is described below. An overview of each step can be seen in Figure 2.

Step 1 of the framework asks: **Is the novel farming species likely to be sentient?** Of the 13 respondents, 11 said *yes* and two said ‘*no*’ (collared peccary Respondent 2 and black soldier fly Respondent 2). The explanation given for the collared peccary not being sentient was ‘*it is not an endangered species*’, suggesting that in this case, the definition of sentience had been misinterpreted. Black soldier fly Respondent 2 explained that they thought that the probability of sentience was high enough to warrant concern, and that a precautionary principle should therefore be applied for the farming of this species.

Step 1a of the framework was posed only to the two respondents who had reported that their chosen species was non-sentient (collared peccary Respondent 2 and black soldier fly Respondent 2). This step asks: **Are there likely to be significant negative human or environmental impacts to farming the novel species?** Both responses were negative, with the explanation for the collared peccary being ‘*it is not an endangered species, no problems with pollution, easy to feed, resistant to diseases*’, and for the black soldier fly, ‘*BSF could be used as waste processors, making them valuable parts of a circular food system. If they are not sentient, it would probably be good to farm them, assuming concerns about methane from insect frass can be addressed*’. No further feedback or suggestions were made by respondents about this step of the framework. According to the framework, the answers provided by these two respondents would have then led them to the outcome that farming these non-typical species may be acceptable.

Step 2 asks: **Is there sufficient knowledge of the biological habitat, behaviour and diseases of the novel species to estimate the impact of farming on welfare?** Eleven respondents experienced this step, with eight answering ‘*yes*’ and three saying ‘*no*’ (black solder fly Respondent 1, ostrich, crocodilian). Those answering yes pointed to evidence from both wild and captive populations as being useful. There was considered insufficient evidence in general for black soldier flies and crocodilians, whereas for ostrich, a wider concern about inappropriate extrapolation from other species was cited. No additional considerations or missing components that the respondents felt would be important to guide people through this step of the framework were provided. All 11 respondents were taken to Step 3 (including the three respondents who answered ‘*no*’ to Step 2, and to whom Step 2a of the framework would therefore have been relevant, which states that more research should be conducted to gain sufficient understanding of the species).

Step 3 asks: **Is it likely the novel species would be farmed in such a way that would provide excellent lifelong welfare and a humane death?** Here, 10 of the 11 respondents said ‘*yes*’, whilst one (black soldier fly Respondent 1) said ‘*no*’, stating ‘*It is possible that high welfare farming could occur—but it is unlikely to happen given cost constraints in the feed sector*’. Whilst this respondent felt humane slaughter could be achieved for low cost, they raised concerns about the costs of providing high welfare for adult flies and development of health problems seen in other forms of insect farming such as for silk. The other respondents tended to cite their experience that either the biology of their chosen species supported the possibility of high welfare farming, or that existing farms already provided high welfare set-ups, notwithstanding that some welfare challenges were described, such as decapitation as a slaughter method for oriental rat snakes, whose brains, it was claimed, can continue to feel pain after slaughter. The black soldier fly respondent was taken to Step 3a of the framework, whilst all others were taken to Step 4.

Step 3a was completed by just one respondent, who had answered no during Step 3 (black soldier fly Respondent 2). This step asks: **Is small-scale farming of a few animals of the novel species likely to substantially improve an exceptional particular environmental or human ethical concern such as malnourishment or severe poverty?** The answer given was ‘*yes*’, with the following supporting information: ‘*It seems like small-scale farming could solve an important human ethical concern—lack of access to adequate protein in the face of growing climate catastrophe. BSFL can be fed on nearly any waste stream, including human waste and food waste that is readily available in rural communities with few other resources. They grow quickly and, if safety details can be confirmed, could serve as a stable source of year-round protein for people without regular access to protein. Further, small-scale farming of this species can reclaim waste, allowing previously underutilised nutrient sources to be turned into animal feed or human food. Thus, the potential of this species to be farmed in ways that help solve human ethical concerns is quite high. In fact, even large-scale farming of this species (insofar as it relates to reclaiming wastes) could be a benefit by reducing climate change impacts of our current agricultural system, helping provide more protein for the world (especially if they were approved for human consumption and adopted as a part of the human diet to replace vertebrate animals), and through bioremediation of the environment*’. By answering yes, this respondent’s framework outcome was that the species may be acceptable for farming.

Step 4 was completed by ten respondents. It asks: **Are there likely to be significant negative human or environmental impacts to farming the novel species?** Two answered ‘*yes*’ (ostrich and collared peccary Respondent 1), and therefore reached the end of the framework questions, with the outcome that the species should not currently be considered suitable for farming. However, the comments appeared to suggest that these were erroneous responses, as the collared peccary response described the excellent productivity, reduced greenhouse gas emissions, and lack of deforestation as key attributes of farming, and the ostrich response stated that any environmental challenges were not important in comparison to providing economic viability for rural communities. The other eight responses tended to report the low risk to human health, adaptation to local climate and feedstuffs, and the low environmental impact, even for the snakes, who are noted to be obligate carnivores and hence need to be provided with prey for food, but efficient due in part to being poikilothermic. These respondents were taken to Step 5 of the framework.

Step 5 of the framework asks: **Is farming the novel species a preferable alternative to existing farming methods to meet human nutritional needs?** Eight respondents reached this step of the framework, and all answered ‘*yes*’, with the outcome that these species may be acceptable for farming. It was suggested for capybara that they could provide a cheaper animal protein source for food-insecure people, and that agouti and American bison meat may be particularly healthy. In addition, bison were described as able to utilise poorer quality pastures than typically farmed cattle. It was noted that reptiles are often overlooked for food, but that many people already eat them, and that they may provide cheap food, or in the case of scorpion mud turtles, farming may help prevent overhunting or poaching. In addition, farming crocodilians in tropical regions where cattle do not thrive was posed to be especially advantageous.

### 3.4. Further Questions

After the step-by-step framework questions, respondents were asked how helpful they thought the farming of their chosen species was for ensuring human food security. The majority (n = 7) said ‘*extremely helpful*’, and nine respondents expanded their answers. In summary, capybara farming (‘*extremely helpful*’ and ‘*somewhat helpful*’) was described as providing both food and income for local communities, and aligning with regional ecology and cultural practices, whilst agouti farming (‘*extremely helpful*’) was seen as an important source of otherwise scarce animal protein. Oriental rat snake farming (‘*extremely helpful*’) was seen to offer a cheap, easy, and safe alternative to the chicken and pork industries, which face increasing risks from antibiotic resistance and infectious zoonotic disease outbreaks. Furthermore, this snake meat is accepted and enjoyed for its taste, texture, and nutrition in Asian culture. On the other hand, python meat (‘*somewhat helpful*’) is reportedly eaten in local communities mostly during periods of swine or avian flu outbreaks, and likely because it is primarily farmed for its skin. Ostrich farming (‘*extremely helpful*’) was seen as supporting food security indirectly by boosting local livelihoods. Bison meat (‘*somewhat helpful’*), whilst in high demand via successful farming businesses in the USA, was highlighted as small scale compared with existing farming practice. BSF 1 (‘*extremely helpful*’), could contribute to human food security, but only if farmed locally and fed with food waste, thus replacing other livestock protein. Finally, crocodilian farming (‘*somewhat helpful*’) was considered a commercially viable solution to food security, providing potential regional benefits to ecosystems with decreased pressure on transport and fossil fuel consumption. Without additional explanation, collared peccary was considered either ‘*extremely helpful*’ or ‘*neither helpful nor unhelpful*’ and BSF 2 was considered ‘*neither helpful nor unhelpful*’.

### 3.5. Feedback About the Framework

When asked how applicable the respondents considered the ethical framework for farming non-typical species to be across cultures based on their own experience, seven said ‘*extremely applicable*’, five said ‘*somewhat applicable*,’ and one said ‘*neither applicable nor unapplicable*’. Of those who expanded their feedback about application across cultures, the framework was described as ‘*neutral, free of emotion… can be applied across any culture*’, ‘*very useful for codifying animal welfare in novel production systems*’, and ‘*the questions are clearly presented, indicating no preconceptions or biases*’. Some respondents suggested areas they felt were lacking in the framework, especially acknowledging the value of farming to indigenous people and their contribution of knowledge about non-typical species: ‘*the true impact and value for rural communities are not considered*’, ‘*[indigenous] persons understand the captivity and use of these animals better than most scientists*’, and ‘*impossible to interpret [the framework] without taking into account differing ideologies and worldviews*’.

In relation to whether they would like to see anything additional in the framework, four respondents said ‘*yes*’ and nine said ‘*no*’. Suggested additions were: assessment of the effect that farmed animals have in rural communities; consideration of the cultural significance of the animal to indigenous people; more opportunity for discussion about whether farming a novel species (such as insect farming for animal feed) props up more ‘*clearly objectionable forms of farming*’, and reflection on the mandate for the questions. When asked if there was anything redundant in the framework, 11 answered ‘*no*’ whilst two said ‘*yes*’. Regarding Step 2a of the framework, it was suggested that practical experience comes before scientific evidence and that many new species would not be farmed if ‘*we always waited until science had a particular level of answers about a specific species*’ and that farming determined questions for science to answer. Another respondent felt that the framework represented a ‘*1st world perspective and over anthropogenic relevance allocated to animals*’.

Respondents were asked if they could provide examples of ‘*small-scale farming of a non-typical species that may improve an exceptional environmental or human ethical concern (e.g., malnutrition, extreme poverty), even if there are significant welfare implications for the animals involved*’ (Step 3a of the framework). Ten respondents suggested that either their chosen species or another were exemplars that supported human sustenance or environmental concerns, yet most appeared to endorse farming without addressing welfare. One respondent suggested that farmed animal welfare is community-defined, and not for concern over and above human survival. With relevance to the ethical dilemma highlighted in Step 3a, one respondent mooted that feeding black soldier fly human faeces in certain circumstances to promote human health may fall into this category because fly mortality increases in this situation. They also discussed whether feeding plastics that would otherwise not biodegrade to mealworm larvae may also be acceptable, even if welfare was poor. We also asked whether there were other non-typical species being farmed that were not yet in the scientific literature, and it was suggested that as well as Anseriformes and insects, there is significant expansion for the experimentation of farming a whole variety of reptile species in Asia, and that, for example, *Elaphe carinata* (King rat snake) may prove more efficient than pythons.

Four responses (who had applied the Burmese python, Oriental rat snake, American bison, and ostrich to the framework) included further reflections about wildlife farming from their own experience. In summary, the farming of snakes and other reptiles was considered as having significant potential, with fewer risks to welfare than avian and mammal farming, alongside more efficient and sustainable use of resources. However, it was highlighted that further research was needed to develop evidence-based standards to inform the management and production of farmed reptilians. The American bison respondent described their visits to farms for many wild animal species, noting that by raising animals in their natural environment, less human intervention was needed and that more welfare-friendly, intentional approaches resulted across various aspects of husbandry. Finally, the ostrich respondent did not describe their own experiences but felt that the aims of our research were remote from the origins of farming wild animal species.

### 3.6. Proposed Revisions to the Mullan et al. [4] Framework

As a result of the responses received, we made small changes to the framework (also see Figure 2 where additions to the text appear in blue and removed words have been struck through). It seemed apparent that either the definition or respondent understanding of sentience was unclear in some instances, leading to potentially erroneous responses (see Section 4). Therefore, a brief definition of sentience is now provided in Step 1 of the model, alongside the suggestion of adopting a precautionary principle when desirable. Another change involved Step 2a, which now appears in an amber box to reflect the outcome that farming is unsuitable if insufficient knowledge is held about the species, but with a broken blue line leading back to the rest of the framework to represent the fluidity of farming practice considering emerging evidence. Further amendments include the text in the green, amber, and red outcome boxes, which again indicate the nature of evidence-based farming, where new research and alterations in practice may change the outcome of a species being run through the framework. Small additions were also made to the background supporting information originally outlined by Mullan et al. [4]. These relevant considerations are designed to guide respondents through each step of the framework and now more explicitly emphasise farming non-typical species in relation to cultural significance, practical experience, and rural and/or indigenous communities (see Supplementary Materials).

## 4. Discussion

This first study surveying academics working in the field of farming non-typical species primarily to support human food production yielded some detailed and insightful information about the species discussed as well as providing some validating evidence for minor modifications to the published ethical framework [4]. This is a small research field, and it proved challenging to identify suitable respondents to approach, hence allowing snowball sampling to try to increase the response rate. Despite this limitation, of those we approached directly, the 13 survey completions represented a 25% response rate (although it was possible for an individual person to submit more than one response for different species, which we believe happened for the two snake species). Although a small survey, the response rate was similar to the 24% gained by [1] for interviews of academics studying wildlife farming. As in [1], our respondents were ‘key informants’ with deep and specialised knowledge, and in most cases, more than ten years of experience in their relevant field. Three of these respondents chose to leave their contact details in case of future research or communications.

The framework challenges people to consider the ethics and sentience of farmed wild species, where there are otherwise limited discussions. However, for some survey respondents, there appear to have been potential barriers to engagement with the framework, where it seemed that the intention behind some questions was misunderstood, impacting on how the framework was experienced. In Step 1, two respondents answered that their species of choice was not sentient (collared peccary 2 and black soldier fly 2), and were thus taken to Step 1a where the framework questions ended. One of these instances suggests either an unfamiliarity with the terminology or a language barrier, given that the explanation for a lack of sentience was that the species was not endangered. The other respondent described precautionary reasoning about sentience ‘*The likelihood of sentience strikes me as high enough to warrant the application of the precautionary principle*’ yet still selected non-sentience. As a result, to improve construct validity of the framework, the term sentience has now been more explicitly defined in Step 1 in our revised iteration of the framework. In Step 4, two respondents expressed that there were likely to be significant negative human or environmental impacts to farming their chosen species (collared peccary 1 and ostrich), and the framework therefore ended there with farming deemed unsuitable for these species. However, the collared peccary respondent described only environmental benefits compared to beef cattle farming, suggesting an erroneous response and that this species may well be suitable for farming. The ostrich respondent did not describe any significant negative impacts of farming this species, so this may also be an erroneous answer.

These examples highlight a limitation of completing the framework by online survey format. Other options could be via structured interviews, where an interviewer could reframe a misunderstood question, or at an organisational or collaborative level between experts of the same species. Furthermore, individual responses are limited by the extent of their own knowledge and experience and/or what they choose to share. For example, regarding the total numbers of ostrich farmed globally, the respondent for this species only discussed South African production, which accounts for 70% of the world’s ostrich products [21]. One possibility could be to clearly require certain attributes of users, such as sufficient knowledge of animal welfare and/or animal ethics, or to include an option for respondents to estimate their degree of certainty about their answers. It was interesting, yet perhaps expected, that individual key informants applied the framework to the same species differently, particularly in the case of black solider fly sentience. Conversations and collaborative reasoning between key informants could be harnessed to inform the ultimate outcome of the framework for a given species’ farming suitability. Another possible limitation of the study population is the self-selection of key informants, where the survey may have attracted people who were predominantly keen to promote or support the farming of non-typical species: 10/11 respondents answered ‘*yes*’ about their chosen species being farmed with excellent lifelong welfare and a humane death. However, it is recognised that the wording in Step 3 which relates to welfare, was prospective, so the respondents may have also been appraising possible as opposed to actual welfare.

Other potential influences on completion of the framework include the respondents’ psychological and cultural influences and orientations. For example, if a respondent is either unaware of an existing evidence base about animal welfare science or sentience or has a negative regard for such science, then pre-existing barriers may impact their engagement with the framework and/or overall questioning of the suitability of farming a species under discussion from this ethical standpoint. One respondent provided feedback that revealed preconceptions about the framework and its creators, questioning whether our ‘*own motivations of activism [are] more important than [rural livelihoods]*’, with ‘*poor understanding of the impact and necessity of farming enterprises in rural ecology*’, suggesting that ‘*we drive destruction of communities to satisfy our own hypothesis*’. We considered this feedback at length and acknowledge the situating of researchers as an influence on scientific studies [22]. In response, we highlight our consideration of the complex and variable impact and value of farming non-typical species across unique contexts and communities, and in particular, Step 3a of the framework, which seeks examples of farming that substantially improve a human or environmental ethical concern, prevailing over animal welfare. Ideally, as well as applying an ethical framework, more intensive field research would be conducted per species, requiring direct assessments of the complex political, economic, cultural, social, legal, and ecological conditions (factors described by Fennell [23], in relation to the ethics of animal-based tourism and Coals et al [24] in relation to breeding lions for traditional medicine) of different forms of farming non-typical species. Given the limitations discussed, we consider the study to have been only moderately effective in stress testing the framework, but nevertheless a useful contribution to this emerging field of practice and study.

Data from additional survey questions provided context to the framework responses in this study, including about the scale of farming per species. The estimated number of farmed wild animals over the past ten to twelve years sits at almost one thousand million, yet the reality is likely far higher [2]. Our findings reflect the consensus that farming many non-typical species is rising [1], with only capybara reported by key informants as declining, six species to be increasing, and four to be unchanged. Reportedly low numbers for some species (e.g., scorpion mud turtle with 1–10 farms, but on the increase) suggest that some farming practices may be in their infancy, very niche, or that there is no current driver for their expansion. Additional data on the type of farming systems used for each species revealed that this varied from free-ranging in their natural environment to more intensive management including cages or pens, or trays for insects. Measures of welfare are beyond the scope of the framework, but this may be an area that warrants further attention in field research, where living a good life [25] may not be possible in intensively managed environments, in which case Step 3 of the framework may be violated.

Whilst the framework so far has been primarily applied to species farmed for human consumption, future studies could investigate the farming of species for other primary purposes (e.g., Traditional Chinese Medicine, luxury fashion, animal feed, the pet trade) and target species for new farming proposals such as cephalopods. The ethical framework has extensive potential applications and could be used both proactively and reactively. For example, governments might put species through the framework to decide policies on their farming; investors might use it to decide what aspect of farming to trial or invest in; researchers and farmers may wish to evaluate existing or potential systems; organisations (including non-governmental organisations), for example, could apply the framework to decide whether to provide support for government foreign aid investment. With research on non-typical farming still sparse, future applications of the framework may lead to additional revisions and considerations for dissemination, not only of the framework, but also for the evaluation of its outcomes across different species. Future versions may also include language translations or other adaptations for use with indigenous stakeholder communities. An open access portal enabling application of the framework alongside data collection about non-typical species farming may provide useful two-way progression of knowledge.

## 5. Conclusions

The huge and increasing numbers of non-typical species being farmed for food and other products warrants consideration of their welfare, the interests of farmers, impact on conservation, and other morally relevant factors. Despite the small number of respondents, this exploratory study to assess the ethical acceptability of such farming by 13 academic key informants with knowledge of different species has provided an opportunity to refine the construct validity and usefulness of the framework prior to future applications with other stakeholders and species, where further refinement may also be necessary. As well as responding with information about the animals, some key informants stressed the importance of understanding the human contexts in which the farming is occurring. We recommend further research to more fully explore the ethical acceptability of farming non-typical species on a case by case basis. Ultimately, this framework may provide an iterative and innovative process to help bridge the gap between existing and future practices and policies surrounding the farming of all new and non-typical species, and the complex ethics of human–animal–environment relationships that influence how we should act.

## Figures and Tables

**Table 1:** Self-described research discipline, time spent researching, and geographic focus of the respondents.

Response	Research Discipline	Length of Time Researching Farming Non-Typical Species	Geographic Research Focus
1	Not provided	10+ years	South America
2	Not provided	10+ years	South America
4	Farming neo tropical wild animals for sustainability conservation and use	10+ years	South America
3	Animal science and production	10+ years	South America
5	Applied herpetology, biodiversity conservation	10+ years	Africa, Asia
6	Applied herpetology, biodiversity conservation	10+ years	Africa, Asia
7	Entomology	1–5 years	Africa, Asia, Europe, North America
8	Mammalogy	10+ years	South America
9	Philosophy	1–5 years	Europe, North America
10	Bison and wapiti (*Cervus elaphus*) production	10+ years	North America
11	Wild animals: production and conservation	10+ years	South America
12	Veterinary production animals	10+ years	Africa
13	Behaviour, physiology, neurophysiology, microbiology	1–5 years	Africa, Australia/Oceania

**Table 2:** Background information of the species of choice as reported by the respondents (one row per response).

Chosen Species	Prevalent Driver(s) for Farming	Approx. Total Number of Farms	Approx. Total Number of Animals in All Countries	Approx. Total Annual Slaughter	Farming Increasing/Declining?	Continents Farmed	Countries Farmed	Farming System(s)
Capybara (*Hydrochoerus hydrochaeris*)	Other: Nowadays, food security has taken a back seat as capybara populations have increased greatly and there is a conflict between animal and human coexistence, mainly due to the transmission of spotted fever by ticks hosted by capybaras.	51–100	60–100	1 to 50	Declining	South America	Brazil, Argentina, Venezuela	In Brazil, capybaras are mostly raised in captivity, in paddocks. In Venezuela, they are wild harvested.
Capybara (*Hydrochoerus hydrochaeris*)	Food security, human sustenance, Economic, fashion industry	11 to 50	100–500	Unknown	Declining	South America	Brazil, Argentina, Venezuela, Colombia, Peru	Farms or specialised facilities—diet, health, and breeding are managed, producing primarily for meat and leather. Allows for selective breeding, enhancing desirable traits in the capybara population. Semi-captive systems—large, fenced areas that mimic their natural habitats but with some level of human intervention. Provides capybaras with more space and a natural diet while still allowing for some degree of management and protection from predators. Semi-captive breeding aims to balance animal welfare with economic production, making it a popular choice among producers seeking sustainable methods. Wild capybaras are also legally hunted in some countries.
Collared peccary (*Dicotyles tajacu*)	Food security/human sustenance, economic	11 to 50	1000–5000	Unknown	Unchanging	South America	Brazil	Most farmers use a semi-confined production system. This is because Brazil prohibits the commercial hunting of collared peccaries and other wild species. Peccaries are kept in paddocks ranging in size from 400 m^2^ to 5 hectares. Males are kept with females at all times and females do not need to be isolated to give birth. These farmers usually feed the peccaries with food resources available in the region, such as by-products from the agricultural production of the property and its neighbours. The young grow up in the same group in which they were born and reach a slaughter weight of between 19 and 22 kg at the age of 10 to 15 months (depending on the type of food they eat).
Collared peccary (*Dicotyles tajacu*)	Food security/human sustenance, economic, conservation, fashion industry, cultural factors, organised wildlife crime	Unknown	Unknown	Unknown	Unknown	South America	Brazil, Peru, Bolivia, Argentina	Intensive and semi-extensive enclosure design, good reproduction behaviour and management. Slaughter techniques based on domestic animals. Easy to feed. Farmed for meat and skin.
Agouti (*Dasyprocta*)	Food security/human sustenance, economic, conservation	11 to 50	5000–10,000	5000–10,000	Increasing	South America	10 different countries, not specified	Intensive farming, captive breeding in cages and floor pens, slaughtered at farm abattoir, fed rabbit pellets and local feedstuff
Burmese python (*Python bivittatus*)	Economic, fashion industry	Unknown	Unknown	Unknown	Unchanging	Asia	Vietnam, Thailand	Exclusively captive including breeding. Small and large scale intensive production and processing not unlike poultry and pig farming. Slaughter involves complete brain destruction by hammer or captive bolt gun. Diet includes waste protein from agri-food chains (e.g., still born pigs and fish skins) and pest rodents harvested from rice fields.
Oriental rat snake (*Ptyas mucosa*)	Food security/human sustenance, economic	500+	Unknown	Unknown	Unchanging	Asia	China, Vietnam	Closed cycle breeding and rearing facilities. Large enclosures housing hundreds of individuals. Fed poultry waste. Mostly live sales to snake markets and direct to restaurants.
Black soldier fly (*Hermetia illucens*)	Food security/human sustenance, economic, other: to support cheaper, and possibly more sustainable, feed options for vertebrate livestock animals. Realistically, few will be consumed directly by humans.	100–500	8–16 billion (under-estimate as industry growing rapidly)	190 billion BSF are slaughtered each year around the globe as of 2020	Increasing	Africa, Asia, Europe, Oceania, North America, South America	U.S., Canada, Australia, UK, many Asian (e.g., TH, CN), EU (e.g., NL), and African nations (e.g., ZA)	Exclusively captive. Larvae are fed substrates of varying quality in small pans or large trays, and are the primary product that is slaughtered; adults, used as breeders, are housed in mesh-sided mating cages or in large greenhouses. Food, and sometimes water, is not provided for adults, which desiccate or starve to death. Slaughter includes oven baking, microwaving, grinding/shredding, boiling/blanching, sun roasting, sand roasting, freezing in air, asphyxiation, freezing in liquid nitrogen. A minority are live-fed to consumer animals.
Black soldier fly (*Hermetia illucens*)	Other: BSF are reared as feed for conventional terrestrial and aquatic livestock.	100–500	1,000,000+	Primary product is not meat (e.g., eggs)	Increasing	Europe, North America	U.S., France	Scientists do not know much about the abilities of BSF larvae, farmed mostly for animal feed. However, it is known that when heated, they thrash around and try to escape and when cut, they roll around very rapidly. They are raised in an organic substrate that they crawl through and eat. Mixed substrates (for instance, grains and vegetable waste) offer a better balance of nutrients and promote heath than simple substrates, and they show preferences for certain foodstuffs. If the temperature or moisture level is too high or low, then many of the larvae die. The method of slaughter depends on the intended use but includes grinding, roasting, microwaving, and boiling and are not stunned prior to slaughter.
American bison (*Bison bison*)	Food security/human sustenance	500+	500,000–1,000,000	50,000–100,000	Increasing	North America	U.S., Canada	Breeding is natural, as artificial insemination is not a commercially viable option. Calving is in outdoor pens or pastures in spring. Primarily raised on pasture and typically finished in large feeding areas (more square footage per animal than a typical beef feedlot). The majority of North American bison are slaughtered in federally inspected slaughter plants in the USA and Canada, some in state or provincially inspected plants and even fewer animals are slaughtered on farm. Bison are very adapted to thriving in cold winter temperatures, and snow. They are not raised indoors, and there are not typically shelters in pastures for them. They have the ability to utilise low quality forage and break down highly fibrous plant matter. Their strong metabolic seasonality coincides with the winter and summer seasons and forage availability.
Scorpion mud turtle (*Kinosternon scorpioides*)	Food security/human sustenance, economic, conservation	1 to 10	500–1000	60–100	Increasing	South America	Brazil	The total area of the breeding facility is 200 m^2^, divided into five environments, according to the phases of zootechnical development of animals: artificial hatchery (6 m^2^), nursery (6 m^2^)—animals up to 50 g; recreate (30 m^2^)—animals over 51 g up to 250 g; reproduction (50 m^2^)—animals over 251 g; quarantine (8 m^2^). The reproduction area has a water tank and a sand area, where eggs are laid. The animals received commercial food (32% crude protein), three times a week, based on 1% of body weight.
Ostrich (*Struthio camelus*)	Economic, fashion industry, other: only agri-enterprise suitable for the environment, with a lot of intellectual property in the community. Complete rural economy built on enterprise	100–500	100,000–500,000	100,000–500,000	Unchanging	Africa	South Africa	Complete commercial integrated and non-integrated production system. Domesticated animals only, no collection form the wild or from wild populations. Free range systems with early production—intensive management interaction and protection against environmental conditions decreasing with time as animals grow older. EU approved welfare and slaughter requirements as well as health status verification. Complete individual traceability
Crocodilian spp.	Food security/human sustenance, conservation, fashion industry	51–100	Unknown	Unknown	Increasing	Africa, Asia, Oceania, North America, South America	Australia, Zimbabwe, U.S., Papua New Guinea	Very variable depending on species of crocodilian—alligators and caimans are captive bred to a greater extent than Nile and saltwater crocodilians. Hatching is in artificial hatcheries, with strict temperature and humidity control. Animals are group housed as hatchlings and growers. Pens have basking areas, ponds and hides. The aggressive species (*C. porosus*) may be finished in single pens with a water body, basking shelf, and hide. Slaughter is captive bolt followed by pithing.

## Data Availability

The raw data supporting the conclusions of this article will be made available by the authors, without undue reservation.

## Supplementary Materials

The following supporting information can be downloaded at: https://www.mdpi.com/article/10.3390/ani16101519/s1. Figure S1: Sentiocentric ethical framework revised from original (Mullan et al., 2024 [4]) in response to survey feedback. Supplementary Table S1: Revisions to the original explanatory text (Mullan et al., 2024 [4]) of relevant considerations that accompany the revised ethical framework. Survey S1: Wildlife farming ethical framework survey.
