# Supplementary material for: To Farm or Not to Farm? Pilot Testing a Sentiocentric Ethical Framework for Farming Non-Typical Species
Source: Animals (Basel). 2026 May 15;16(10):1519. doi: 10.3390/ani16101519 (PMC13203815; doi:10.3390/ani16101519)
Supplement: Supplementary file 1 [file animals-16-01519-s001.zip › animals-4233307-supplementary.pdf]

Supplementary Material

Figure S1 Sentiocentric ethical framework revised from original (Mullan et al., 2024 [4]) in response to survey feedback

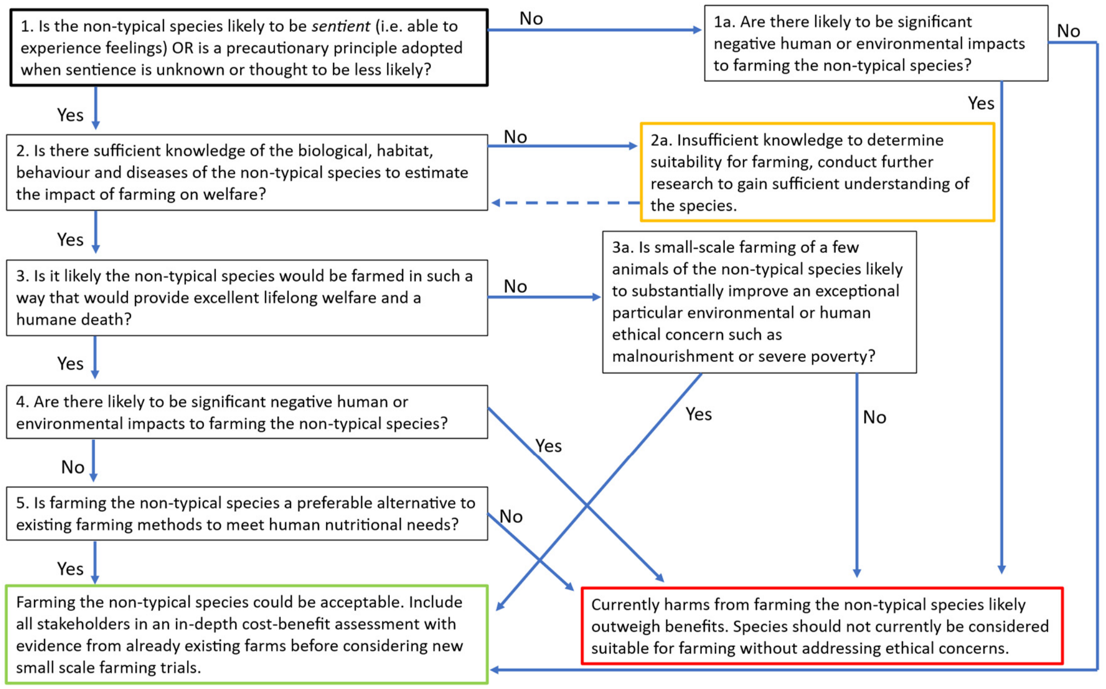

Table S1 Revisions to the original explanatory text (Mullan et al., 2024 [4]) of relevant considerations that accompany the revised ethical framework

**Framework steps with relevant considerations to support completion of the ethical framework for farming non-typical species**	
**Framework step with revisions in blue**	**Relevant considerations (Mullan et al., 2024) with revisions in blue may include:**
Step 1: Is the non-typical farming species likely to be *sentient*? (i.e., able to experience feelings) OR is a precautionary principle adopted when sentience is unknown or less likely?	Is the species a vertebrate, cephalopods or decapod crustacean? For all other species: how strong is the evidence of sentience? Is there any evidence of lack of sentience? What is the quality of that evidence? How does the likelihood of sentience affect any application of a precautionary principle for this species? Consider the plausibility of sentience where there is a lack of current evidence.
Step 1a: If the species is not likely to be sentient, are there likely to be significant negative human or environmental impacts to farming the non-typical species?	Are there any likely human safety or public health implications for farming the species? Are there aspects of farming that are likely to result in high greenhouse gas emissions, loss of biodiversity or pollution? Where does the feed for the animals come from? Can it be grown locally with low emissions? Can the species be farmed in harmony with the local environment and resources? Are there disease risk implications for local wild animals? Will the removal of native animals be required to set up farms have significant impacts on local ecosystems?
Step 2: Is there sufficient knowledge of the biological habitat, behaviour and diseases of the non-typical species to estimate the impact of farming on welfare?	What evidence is available on key biological aspects of the species, including practical experience and scientific knowledge? What is the quality of this evidence? Where would it sit on a relevant hierarchy of evidence? Where is the evidence derived from (e.g. wild animals, which may be sparse, or captive animals which may have biases resulting from captivity)? Are there relevant biological aspects for which we have little, or poor quality, knowledge? How well would we be able to 'fill in' any biological gaps using information from closely related species?
Step 3: It is likely the non-typical species is being or would be farmed in such a way that provides excellent lifelong welfare and a humane death?	How likely is it that the biological needs are being met or would be easily met with regard to diet, environment, social structure and daily activity? How likely is it that positive welfare experiences are being or could be integrated into the farming system? Is it likely we can or could humanely and safely kill the species? How likely is high welfare farming, considering any practical and economic constraints? Is this species farmed already? What is the welfare of those animals?
Step 3a: Is small-scale farming of a few animals of the non-typical species likely to substantially improve an exceptional particular environmental or human ethical concern such as malnourishment or severe poverty?	What is the exceptional environmental or human ethical concern (including cultural factors) that could trump animal welfare? Are there preferable alternative ways to resolve that concern without farming non-typical species in a low welfare way? How can the animal welfare impact be limited? For example, reducing the number of animals affected, employing a time limitation until preferable systems are in place.
Step 4: Are there likely to be significant negative human or environmental impacts to farming the non-typical species?	Are there any likely human safety or public health implications for farming the species? Are there aspects of farming that are likely to result in high greenhouse gas emissions, loss of biodiversity or pollution? Where does the feed for the animals come from? Can it be grown locally with low emissions? Can the species be farmed in harmony with the local environment and resources and local communities? Are there disease risk implications for local wild animals? Will the removal of native animals be required to set up farms and will this have significant impacts on local ecosystems?

Step 5: Is farming the non-typical species a preferable alternative to existing farming methods to meet human nutritional needs?	What is the impact of the relevant existing comparator farming systems for the specific human population? i.e. what species are currently eaten, and from what systems? Would the non-typical farming system deliver benefits to the local population over existing systems, particularly considering socio-economic, geographical or other limitations for accessing existing animal protein? Does farming this species preserve human culture? Are there particular regions, areas or people who are particularly likely to benefit from farming non-typical species?
