# Supplementary material for: To Farm or Not to Farm? Pilot Testing a Sentiocentric Ethical Framework for Farming Non-Typical Species
Source: Animals (Basel). 2026 May 15;16(10):1519. doi: 10.3390/ani16101519 (PMC13203815; doi:10.3390/ani16101519)
Supplement: Supplementary file 1 [file animals-16-01519-s001.zip › Supplementary material_Survery S1.pdf]

Wildlife farming ethical framework survey

Survey Consent Form

Research project title: International partnership promoting sustainable use of natural resources: an ethical exploration of farming non-typical (wild) animals.
Research contacts: Dr Helena Hale & Professor Siobhan Mullan

I have read and understand the information sheet for the study and have had the opportunity to consider the information *

◯ Yes

I can ask any questions by contacting the researcher before taking part, or with any future questions *

◯ Yes

I am voluntarily taking part in this project and I can withdraw at any point during the survey *

◯ Yes

I understand that information may be published under the conditions stated in the participant information *

◯ Yes

I don't expect to receive any benefit or payment from participation *

◯ Yes

If I have any concerns about this research or the way it is being conducted, or if I wish to make a complaint I may contact the Ethical Committee at University College Dublin *

◯ Yes

I agree to take part in the above study *

◯ Yes

At the end of the survey, I will have the option to be contacted for further research if I choose to provide my email address. This will not correspond to my survey answers and my participation shall remain anonymous. *

◯ I understand I do not have to provide my email address but if I choose to do so, it will not be linked to my anonymous survey response

Your expertise

The following questions are about your expertise in relation to the farming of novel / non-typical (wild) species, primarily reared for food. This includes species where there is not widespread adoption of farming, even if they have a long history of being farmed (e.g., guinea pigs). Other uses may co-occur, such as farming for conservation or luxury cosmetics, the fashion industry, traditional medicine or the pet trade, but would not be the sole or main output (e.g., do not include bears farmed for bile).

Please state your main research discipline(s) *

[ ]

How long have you been researching aspects relating to the farming of non-typical species? *

- ○ 1-5 years
- ○ 6-10 years
- ○ 10+ years

What is the geographical region(s) of your research on wildlife farming? *

- ☐ Africa
- ☐ Antarctica
- ☐ Asia
- ☐ Australia/Oceania
- ☐ Europe
- ☐ North America
- ☐ South America

Please focus on ONE species that you are most familiar with in relation to wildlife farming when answering the following questions. This should be a species that is farmed primarily for human consumption, where wildlife conservation or use of the animal for luxury cosmetics or fashion, traditional medicine or the pet trade may co-occur but would not be the sole or main output.

Please note, you can complete the survey more than once, if you would like to answer about more than one species.

Please state your chosen species *

In which continent(s) is this species mostly being farmed? *

- [ ] Africa
- [ ] Antarctica
- [ ] Asia
- [ ] Australia/Oceania
- [ ] Europe
- [ ] North America
- [ ] South America

Please now state the countries where the species is mostly being farmed *

Is the scale of farming of this species increasing, static or decreasing?

- ○ Increasing
- ○ Static
- ○ Decreasing
- ○ Don't know

Please briefly describe the type of farming system(s) for this species, e.g., exclusively captive including breeding; includes some wild harvesting; enclosure design; slaughter techniques; any other husbandry information *

What are the approximate number of farms across all countries for this species? *

- 1-10
- 11-50
- 51-100
- 100-500
- 500+
- Don't know

What are the approximate total number of animals being kept on farms in all countries at any given time? *

- 1-50
- 60-100
- 100-500
- 500-1,000
- 1,000-5,000
- 5,000-10,000
- 10,000-50,000
- 50,000-100,000
- 100,000-500,000
- 500,000-1,000,000
- 1,000,000+
- Don't know

What are the approximate number of animals being slaughtered annually for meat? (If primary animal product is not meat - e.g., eggs., - please select relevant box) *

- Primary product is not meat (e.g., eggs)
- 1-50
- 60-100
- 100-500
- 500-1,000

- 1,000-5,000
- 5,000-10,000
- 10,000-50,000
- 50,000-100,000
- 100,000-500,000
- 500,000-1,000,000
- 1,000,000+
- Don't know

What do you think is/are the prevalent driver(s) for current farming practice of your chosen non-typical species? Please select as many that apply. *

- [ ] Food security / human sustenance
- [ ] Economic
- [ ] Conservation
- [ ] Cosmetic or medicinal products
- [ ] Fashion industry
- [ ] Cultural factors
- [ ] Organised wildlife crime
- [ ] Other

If you selected 'other', please explain your choice

Questions about the ethical framework (Mullan et al., 2024)

The following questions relate to your chosen species in the context of a proposed ethical framework. The questions will operate to reflect the framework decision-tree, with the framework image (taken from Mullan et al., 2024) provided for information whilst the questions guide you through it.
Please note: you will be taken to the 'further questions' section of the survey at whichever point your answers bring you to the end of the framework, so unless you get all the way to step 5 with your answers, you will not be asked about every section of the model.
At each stage, you will be asked to consider your chosen animal in relation to the framework, as well as some feedback questions about each step.

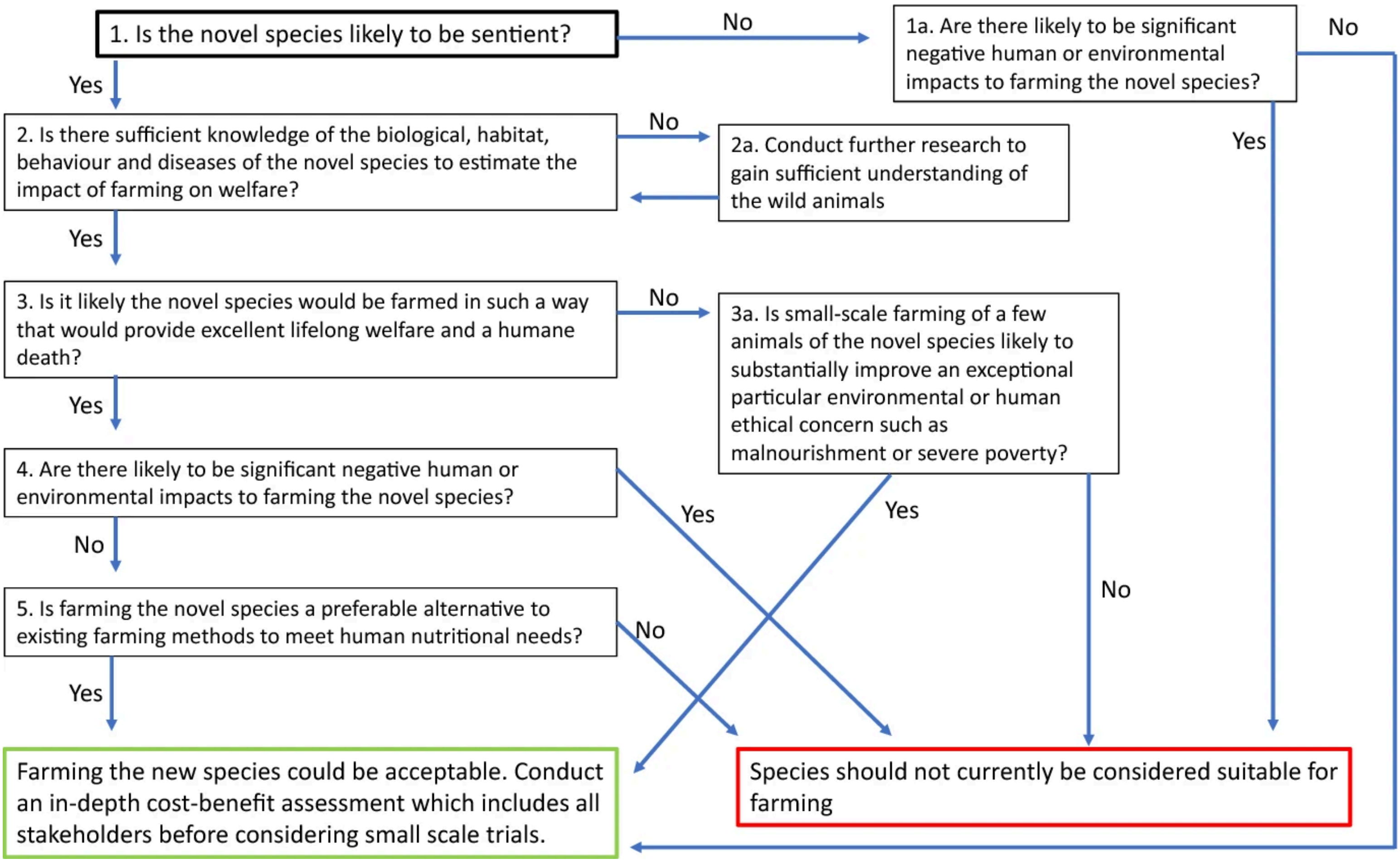

Framework step 1: *Is the novel farming species likely to be sentient?*
(You may wish to consider the following in your response: how strong is the evidence of sentience? Is there any evidence of lack of sentience? What is the quality of that evidence? How does the likelihood of sentience affect any application of a precautionary principle for this species?) *

- ◯ Yes
- ◯ No

Please briefly explain your choice

Please provide any additional considerations or missing components that you feel would be important to guide people through this step of the model?

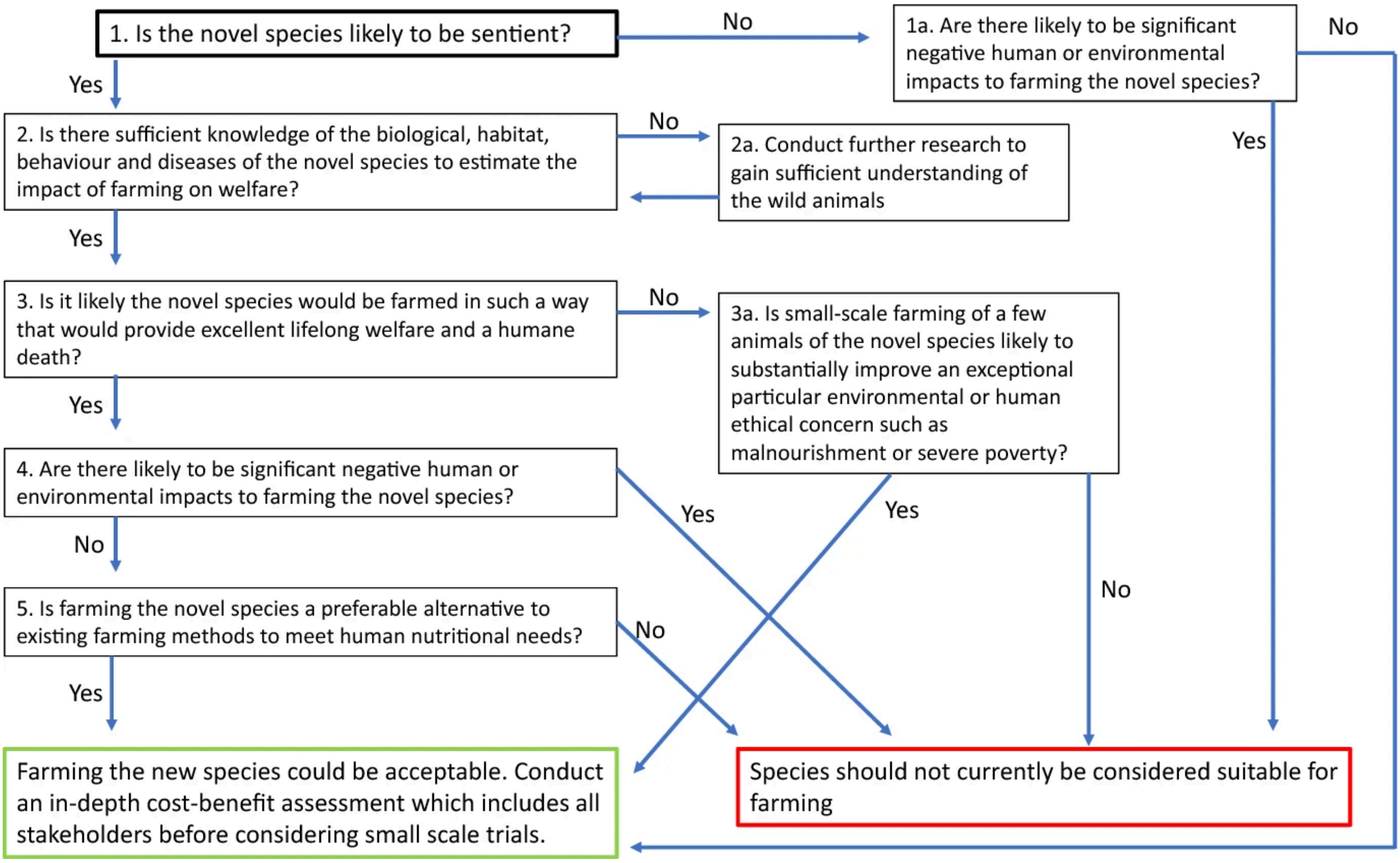

Framework step 2: *Is there sufficient knowledge of the biological habitat, behaviour and diseases of the novel species to estimate the impact of farming on welfare? (Relevant considerations may include: What evidence is available on key biological aspects of the species? What is the quality of this evidence? Where would it sit on a relevant hierarchy of evidence? Where is the evidence derived from (e.g. wild animals, which may be sparse, or captive animals which may have biases resulting from captivity)? Are there relevant biological aspects for which we have little, or poor quality, knowledge? How well would we be able to 'fill in' any biological gaps using information from closely related species?* *

◯ Yes

◯ No

Please give brief details

Please provide any additional considerations or missing components that you feel would be important to guide people through this step of the model?

Framework step 3: *It is likely the novel species would be farmed in such a way that would provide excellent lifelong welfare and a humane death?*

(Relevant considerations for this step include: How likely is it that the biological needs can be easily met with regard to diet, environment, social structure and daily activity? How likely is it that positive welfare experiences could be integrated into the farming system? Is it likely we can humanely and safely kill the species? How likely is high welfare farming, considering any practical and economic constraints? Is this species farmed already? What is the welfare of those animals?) *

- Yes
- No

Please give brief details

Please provide any additional considerations or missing components that you feel would be important to guide people through this step of the model?

Framework step 3a: *Is small-scale farming of a few animals of the novel species likely to substantially improve an exceptional particular environmental or human ethical concern such as malnourishment or severe poverty?*
(Relevant considerations for this step include: What is the exceptional environmental or human ethical concern that could trump animal welfare? Are there preferable alternative ways to resolve that concern without farming non-typical species in a low welfare way? How can the animal welfare impact be limited? For example, reducing the number of animals affected, employing a time limitation until preferable systems are in place.) *

- ◯ Yes
- ◯ No

Please give brief details

Please provide any additional considerations or missing components that you feel would be important to guide people through this step of the model?

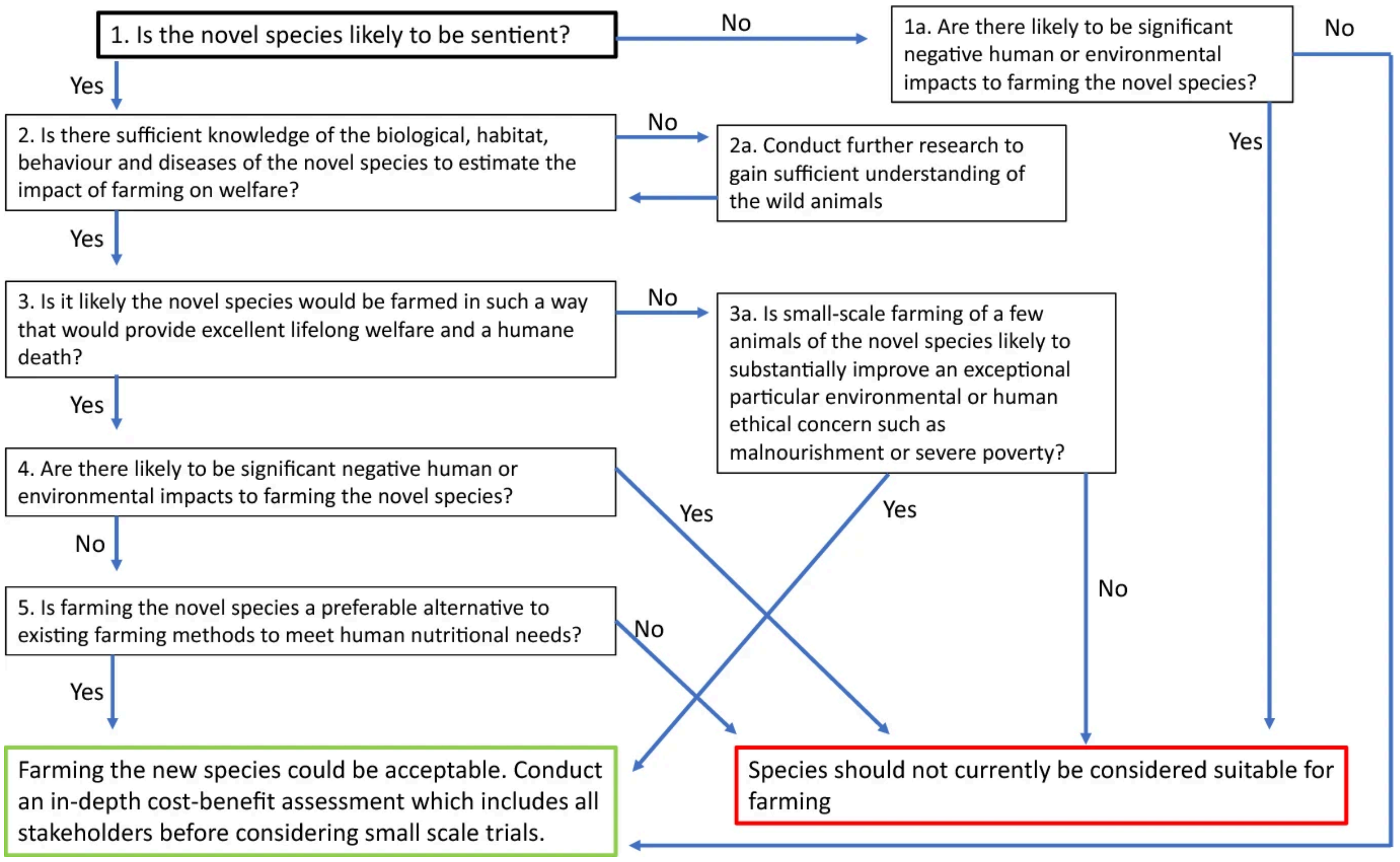

Framework step 1a: *If the species is not likely to be sentient, are there likely to be significant negative human or environmental impacts to farming the novel species? (You may wish to consider the following in your response: Are there any likely human safety or public health implications for farming the species? Are there aspects of farming that are likely to result in high greenhouse gas emissions, loss of biodiversity or pollution? Where does the feed for the animals come from? Can it be grown locally with low emissions? Can the species be farmed in harmony with the local environment and resources? Are there disease risk implications for local wild animals? Will the removal of native animals be required to set up farms have significant impacts on local ecosystems?)* *

- Yes
- No

Please briefly explain your choice

Please provide any additional considerations or missing components that you feel would be important to guide people through this step of the model?

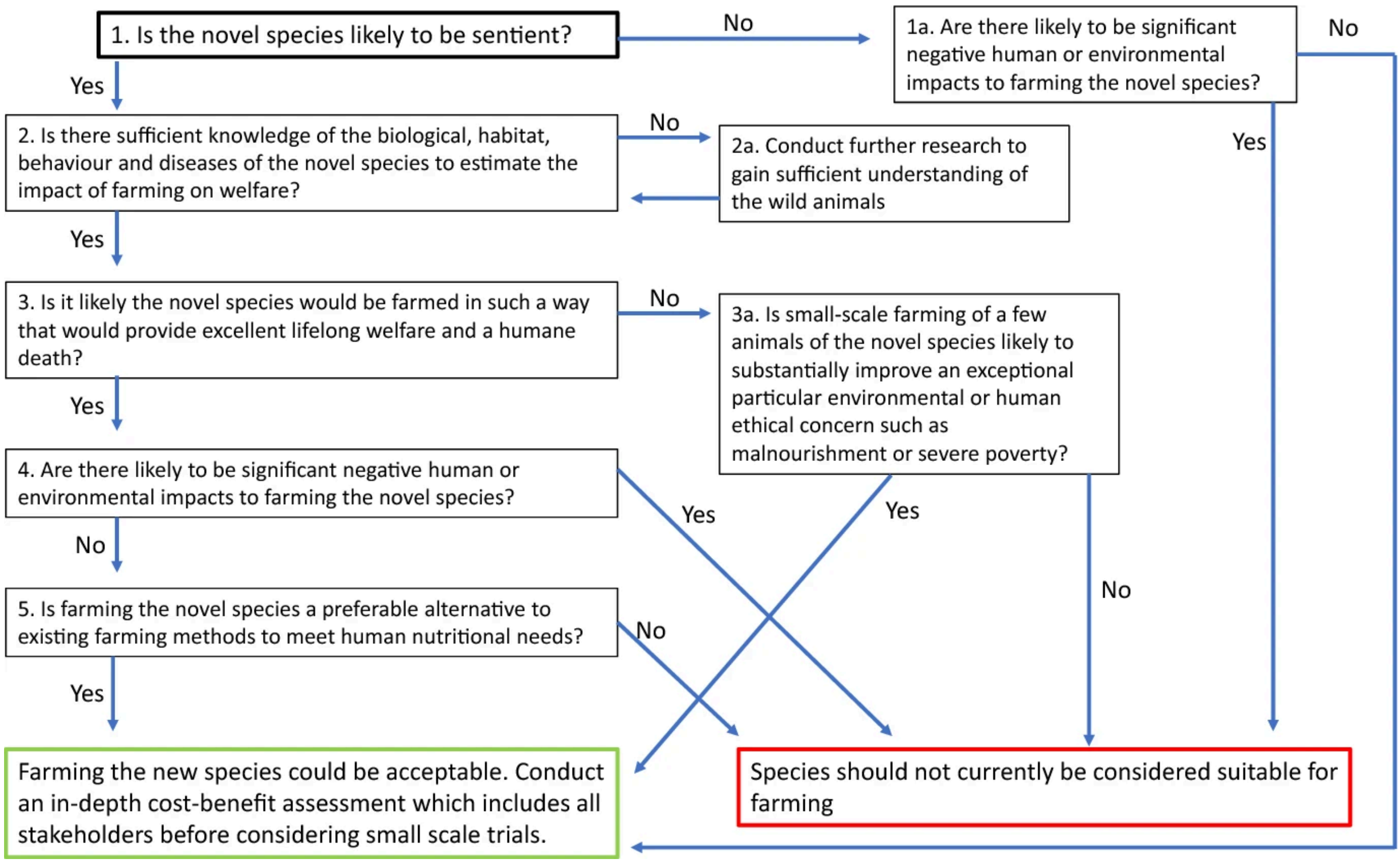

Framework step 4: *Are there likely to be significant negative human or environmental impacts to farming the novel species?*
(You may wish to consider the following in your response: Are there any likely human safety or public health implications for farming the species? Are there aspects of farming that are likely to result in high greenhouse gas emissions, loss of biodiversity or pollution? Where does the feed for the animals come from? Can it be grown locally with low emissions? Can the species be farmed in harmony with the local environment and resources? Are there disease risk implications for local wild animals? Will the removal of native animals be required to set up farms have significant impacts on local ecosystems?) *

◯ Yes

◯ No

Please briefly explain your choice

Please provide any additional considerations or missing components that you feel would be important to guide people through this step of the model?

Framework step 5: *Is farming the novel species a preferable alternative to existing farming methods to meet human nutritional needs?*
(Relevant considerations for this step may include: What is the impact of the relevant existing comparator farming systems for the specific human population? i.e. what species are currently eaten, and from what systems? Would the non-typical farming system deliver benefits to the local population over existing systems, particularly considering socio-economic, geographical or other limitations for accessing existing animal protein? Are there particular regions, areas or people who are particularly likely to benefit from farming non-typical species?) *

- ○ Yes
- ○ No

Please explain your choice

Please provide any additional considerations or missing components that you feel would be important to guide people through this step of the model?

Further questions

You have reached this page because your answer to the previous question has brought you to the end of the model (either because the species should not currently be considered suitable for farming, or that farming the species could be acceptable).
Please now answer the remaining questions that relate to your chosen species and your opinions of the framework (Mullan et al., 2024), which is also visible on this page for your reference.

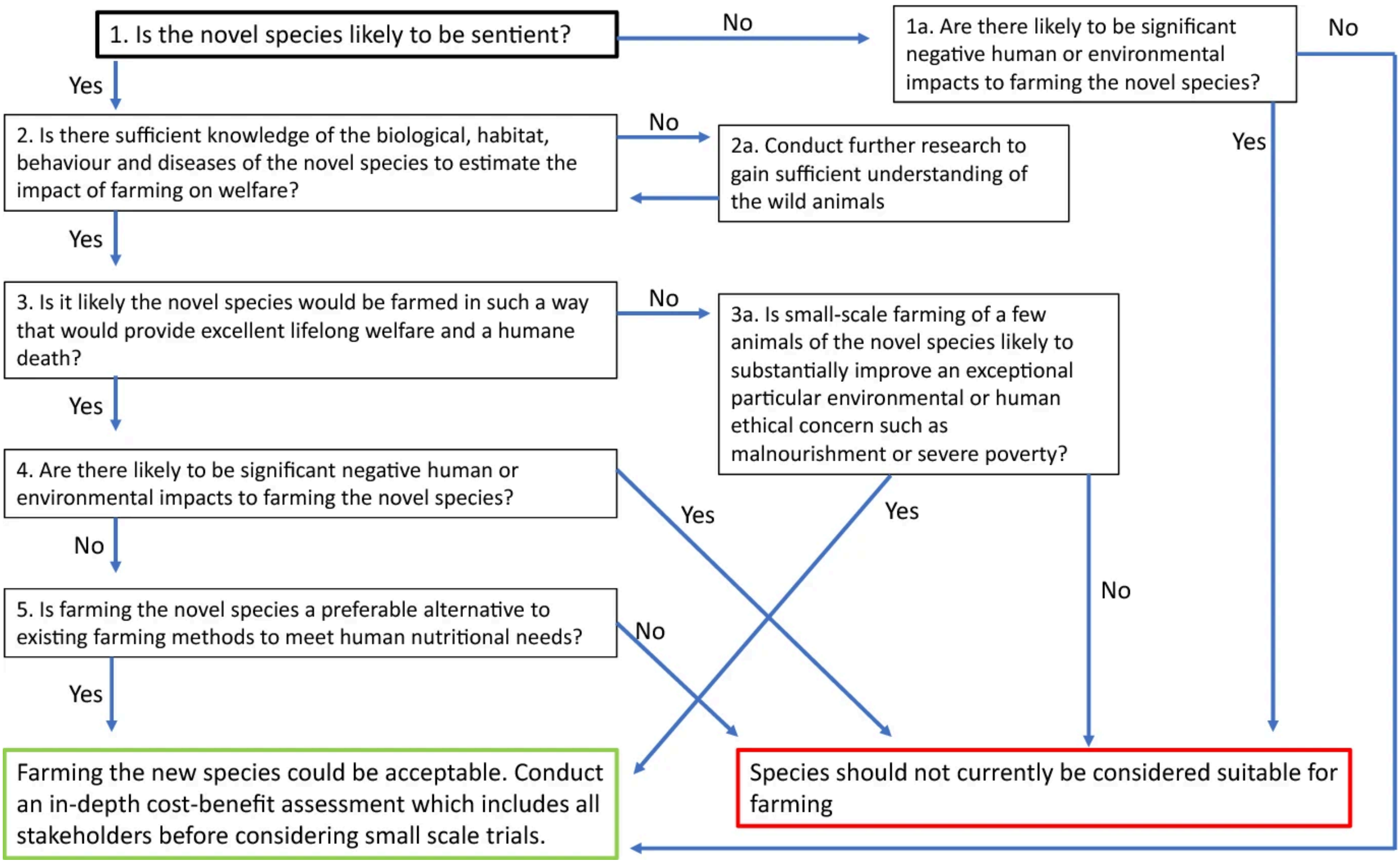

Based on your experience, how applicable do you consider the framework to be across cultures? *

- ○ Not at all applicable
- ○ Somewhat unapplicable
- ○ Neither unapplicable or applicable
- ○ Somewhat applicable
- ○ Extremely applicable

◯ Don't know

Please explain your answer

In your opinion, how helpful is the farming of your chosen non-typical species to ensuring human food security? *

◯ Not at all helpful
◯ Somewhat unhelpful
◯ Neither unhelpful or helpful
◯ Somewhat helpful
◯ Extremely helpful
◯ Don't know

Please explain your answer

Is there anything additional that you would wish to see in the ethical framework for farming non-typical species? *

◯ Yes
◯ No

If yes, please explain your answer

Is there anything that you consider redundant in the ethical framework for farming non-typical species? *

◯ Yes

◯ No

If yes, please explain your answer

We have struggled to identify examples that relate to box 3(a) in the model, where small-scale farming of a non-typical species may improve an exceptional environmental or human ethical concern (e.g. malnutrition, extreme poverty), even if there are significant welfare implications for the animals involved.
Please provide any examples of species or situations that you have experience of, or any comments you may have on this aspect of the framework.

Are you aware of any other non-typical species being farmed, which may not yet be described in peer-reviewed literature? *

◯ Yes

◯ No

If yes, please provide some detail about this/these species

If you answered yes to the previous question, do you think the species would be considered suitable, or unsuitable for farming according to the ethical framework?

◯ Suitable

◯ Unsuitable

◯ Don't know

Please use this space to share anything else that you would like to tell us about in relation to wildlife farming that has not been covered in the survey. For example, you may wish to describe something you have witnessed that is not captured in the published literature surrounding animal welfare and ethics in farming non-typical species, or if you have visited any wildlife farms, how it made you feel.

Thank you

We greatly appreciate your participation in this survey. Please now hit the SUBMIT button.

If you are interested in sharing your email address so that you can be contacted in relation to future relevant research, or would like us to collate your details alongside others to enable contact between an informal community of researchers in this field for possible future discussion or collaboration, please follow this link:
https://forms.office.com/e/Lkc1qwJBqD
Please note: should you wish to complete the survey again for a different species, you can do so by following this link:
https://app.onlinesurveys.jisc.ac.uk/s/svs/wildlife-farming-ethical-framework-survey
